# Evaluating Lumbar Intervertebral Disc Degeneration on a Compositional Level Using Chemical Exchange Saturation Transfer: Preliminary Results in Patients with Adolescent Idiopathic Scoliosis

**DOI:** 10.3390/diagnostics11060934

**Published:** 2021-05-22

**Authors:** Lena M. Wollschläger, Sven Nebelung, Christoph Schleich, Anja Müller-Lutz, Karl L. Radke, Miriam Frenken, Matthias Boschheidgen, Max Prost, Gerald Antoch, Markus R. Konieczny, Daniel B. Abrar

**Affiliations:** 1 Department of Diagnostic and Interventional Radiology, Medical Faculty, University Dusseldorf, D-40225 Dusseldorf, Germany; sven.nebelung@med.uni-duesseldorf.de (S.N.); schleich@radiologie-duesseldorf-mitte.de (C.S.); anja.lutz@med.uni-duesseldorf.de (A.M.-L.); ludger.radke@med.uni-duesseldorf.de (K.L.R.); miriam.frenken@med.uni-duesseldorf.de (M.F.); matthias.boschheidgen@med.uni-duesseldorf.de (M.B.); antoch@med.uni-duesseldorf.de (G.A.); danielbenjamin.abrar@med.uni-duesseldorf.de (D.B.A.); 2 Department of Orthopedics and Trauma Surgery, Medical Faculty, University Dusseldorf, D-40225 Dusseldorf, Germany; max.prost@med.uni-duesseldorf.de (M.P.); markus.konieczny@med.uni-duesseldorf.de (M.R.K.)

**Keywords:** advanced MRI techniques, Idiopathic scoliosis, gagCEST, compositional magnetic resonance imaging of cartilage, disc degeneration

## Abstract

Lumbar intervertebral disc (IVD) degeneration is characterized by structural and compositional changes. This study aimed to assess the glycosaminoglycan (GAG) content of IVDs of patients with adolescent idiopathic scoliosis (AIS) and healthy controls using GAG chemical exchange saturation transfer (gagCEST) imaging. Ten AIS patients (mean age 18.3 ± 8.2 years) and 16 healthy controls (mean age 25.5 ± 1.7 years) were included. Clinical standard morphologic MR images (T1w-, T2w-, and STIR-sequences), to rule out further spinal disorders and assess IVD degeneration using the Pfirrmann score, and compositional gagCEST sequences were acquired on a 3T MRI. In AIS patients, the most distal scoliotic curve was determined on whole-spine conventional radiographs and morphological MRI and IVDs were divided as to whether they were affected by scoliotic deformity, i.e., proximal (affected, aIVDs) or distal (unaffected, uaIVDs) to the stable vertebra of the most distal scoliotic curve. Linear mixed models were used to compare mean gagCEST-values. Over all segments, AIS-patients’ IVDs exhibited significantly lower gagCEST-values than the controls: 2.76 [2.32, 3.20]% (AIS), 3.51 [3.16, 3.86]% (Control); *p* = 0.005. Meanwhile, no significant differences were found for gagCEST values comparing aIVDs with uaIVDs. In conclusion, as a powerful diagnostic adjunct, gagCEST imaging may be prospectively applied to detect early compositional degenerative changes in patients suffering from AIS.

## 1. Introduction

Adolescent idiopathic scoliosis (AIS) is the most common subtype of all idiopathic scolioses with an overall prevalence of 0.5–5.2% and a female predominance [1]. With the disease progressing continuously during the growth of patients, AIS can cause significant back pain, restricted mobility, severe rib deformity, and, ultimately, thoracic insufficiency in advanced stages [2,3]. Scoliotic deformity in AIS leads to remodeling of the intervertebral discs (IVDs) with consecutive morphologic IVD degeneration, frequently associated with lower back pain [4,5,6]. In severe AIS, the treatment of choice is spinal 3-dimensional deformity correction and fusion after skeletal maturity. Over the years, surgical concepts have evolved. Presently, selective fusion [7] is performed by sparing fusion of minor curves, i.e., less affected spinal segments. Short segment fusion leads to less frequent IVD degeneration and pain [8,9]. However, only little is known about preoperative IVD degeneration in patients with AIS [10].

As of today, magnetic resonance tomography (MRI) is the most sensitive imaging technique for the evaluation of degenerative alterations in IVDs [4,11]. While non-degenerative, morphologically unremarkable IVDs exhibit a high signal in T2-weighted (T2-w) sequences due to their high intra-tissue water content [6,12,13], degenerative IVDs have a lower signal due to their decreased water content [4,6]. These signal changes of IVDs are used for the well-known Pfirrmann classification, a validated grading system for the differentiation of non-degenerative and degenerative IVDs based on imaging criteria [12]. However, standard clinical morphologic MRI techniques cannot detect early changes in the nucleus pulposus, i.e., glycosaminoglycan (GAG) depletion, as an early and potentially reversible change indicative of IVD degeneration [14]. GAGs are negatively charged linear polysaccharides that attract water and, alongside the central core protein, make up the proteoglycan (PGs) and tissue solid extracellular matrix components. Primarily in the nucleus pulposus (NP), PGs play a vital role for IVD hydration, thus providing resistance to mechanical stress through high osmotic pressure [4]. Consequently, the PG content of the IVDs provides a quantitative indicator of the structure’s composition and configuration in health and disease, and a potent diagnostic target. In the context of AIS, quantitative evaluation of the PG content may therefore open up diagnostic opportunities in detecting early degeneration and monitoring treatment efficacy beyond mere morphologic evaluation.

The gold standard of compositional cartilage imaging using biochemically sensitive MRI sequences is delayed gadolinium-enhanced MRI of cartilage (dGEMRIC). However, due to recent restrictions on gadolinium-based contrast agents (GBCA) [15], alternative compositional imaging techniques have come into focus that do not rely on GBCA. GAG chemical exchange saturation transfer (gagCEST) imaging is a technique that measures the chemical exchange of hydroxyl (-OH)-protons between GAG and bulk water. To induce the CEST effect, a frequency-specific radiofrequency (RF) pulse is applied to first saturate the solute-bound proton pool at different offset frequencies and then transfer these protons to the bulk water pool through chemical exchange, subsequently reducing its signal. The resultant signal decrease is then used to quantitatively assess the CEST effect at the specific frequency range of GAG, i.e., at 0.9–1.9 ppm [16,17,18]. Previous pioneering studies have demonstrated the close association of CEST effect and GAG content that may be used to quantify IVD GAG content [17,18,19]. Consequently, a solid body of evidence indicates that degenerative and non-degenerative IVDs can be distinguished through quantification of GAG content using gagCEST imaging [19,20,21]. 

As of today, gagCEST imaging has not yet been used for the evaluation of proteoglycan depletion as one of the earliest degenerative changes in lumbar IVDs of patients with AIS. The aim of our study was to compare the GAG contents of (a) lumbar IVDs in AIS patients with healthy controls and (b) of those IVDs in AIS patients that were part of the scoliotic curve and those that were not. We hypothesized that gagCEST values of (a) lumbar IVDs of patients with AIS are significantly lower than corresponding IVDs of the control group and (b) that in AIS patients the same is true for IVDs of the scoliotic curve as compared to those that are not part of the scoliotic curve.

## 2. Materials and Methods

### 2.1. Study Design and Population

This study was designed as a prospective comparative clinical imaging study assessing the proteoglycan content of lumbar IVDs in patients with AIS as compared to healthy controls. Prior to study initiation, approval by the local Ethical Committee (Medical Faculty, University of Düsseldorf, Germany, study number 2019-551) and written informed consent were attained from all participants included, or, in underaged patients, from their legal guardians. 

A total of 11 patients with AIS (mean age 18.3 ± 8.2 years; 7 females, 4 males, disease duration 59.2 ± 102.0 months) and 16 healthy controls (mean age 25.5 ± 1.7 years; 8 females, 8 males) without any previous history of spine disease were prospectively recruited during consultation hours at the Department of Orthopedic and Trauma Surgery (University Hospital Düsseldorf) and among the hospital staff, and were included in this study. Of the 11 AIS patients, 4 were treated with physical therapy only, 5 received bracing therapy, and 2 patients received bracing therapy with subsequent surgery. None of the patients had received surgery prior to study initiation. Table 1 gives details on both study populations.

For all participants, predefined exclusion criteria were a history of spine surgery, musculoskeletal chronic inflammatory diseases, congenital spine conditions including non-idiopathic scoliosis, previous spinal trauma, known IVD extrusion as detected by a potential previous MRI scan or pathological body mass index ranges <18.5 or >30 kg/m^2^. However, due to excessive motion artifacts, one female AIS patient had to be excluded from the gagCEST analysis. Acquired spinal deformities and chronic lower back pain were defined as additional exclusion criteria for the control group. Clinically indicated and independent of this study, all included patients had received standard conventional radiographs (CR) of the whole spine in anteroposterior and lateral projections prior to initiation of the study.

### 2.2. MR Imaging

All participants underwent MR imaging of their lumbar spine on a whole-body clinical 3.0T MRI system (Magnetom Prisma, Siemens Healthineers, Erlangen, Germany). Patients were examined in the supine position and imaged with a 32-channel body and a 24-channel spine matrix coil (both Siemens Healthineers). The imaging protocol consisted of a clinical routine protocol, i.e., T1- (T1w) and T2-weighted (T2w) sequences and a short tau inversion recovery (STIR) sequence, and compositional sequences using gagCEST and water saturation shift referencing (WASSR) imaging. All sequences were acquired in the sagittal orientation. Detailed sequence parameters are given in Table 2. 

### 2.3. MR Image Analysis

For image analysis, two clinical radiologists (DBA and CS with 5 and 8 years of experience in musculoskeletal imaging, respectively), blinded to patient data, independently read the MR images. In case of divergent findings, consensus was arbitrated with the assistance of a third clinical radiologist (SN, 8 years of experience in musculoskeletal imaging). 

All participants’ lumbar IVDs (segments L1/L2–L5/S1) were individually graded on sagittal T2w images according to the Pfirrmann classification, an MRI-based five-step grading system for lumbar disc degeneration that enables distinction of degenerated (grade ≥ 3) and non-degenerated (grade ≤ 2) IVDs based on signal intensity and structure of the NP, disc height and the NPs distinction from the annulus fibrosus (AF) [12]. 

On anteroposterior CR of AIS, the Cobb angle and the apical, end, and stable vertebrae were determined using Cobb’s method [22]. By convention, the apical vertebra is the vertebra with the farthest deviation from the center of the vertebral column, while the end vertebrae are those vertebrae that are most tilted towards the apex of the curve. The latter ones are used to measure the Cobb angle by determining the intersection of a tangent drawn along the superior endplate of the superior end vertebra and of a tangent drawn along the inferior endplate of the inferior end vertebra. The stable vertebra is defined as the first vertebra below the lowest curve which is bisected by the central sacral vertical line [23]. 

In all AIS patients, the stable vertebra was situated in the lumbar spine. To further evaluate the influence of scoliotic deformity on GAG content in IVDs in AIS patients, all IVDs proximal to the stable vertebra were compared to those IVDs distal to the stable vertebra and thus not part of the scoliotic curve. To this end, all IVDs of AIS patients, i.e., L1/L2-L5/S1, were divided into two groups depending on whether they were situated proximal or distal to the stable vertebra of the most distal scoliotic curve. For the sake of readability, IVDs situated proximal or distal to the stable vertebra of the most distal scoliotic curve are referred to as “affected” IVDs (aIVDs) or “non affected” IVDs (uaIVDs). For both groups, gagCEST values were determined and statistically analyzed.

For our study, all gagCEST analyses were performed as before [20,24]. Briefly, gagCEST values were derived using a customized in-house script implemented in Matlab (R2018a, The MathWorks Inc., Natick, MA, USA). By means of a diffeomorphic image registration technique integrated into the fMRLung software (Siemens Healthcare), motion correction was conducted for both WASSR (water saturation shift referencing) and CEST images [18]. Using the WASSR maximum-symmetry algorithm to determine a pixel-wise frequency offset curve, B0-field inhomogeneities were corrected as previously published [21,25]. To this end, pixel-wise frequency offset-corrected CEST-curves were determined and divided by the signal without presaturation (S0) to establish the z-spectrum (Z (ω)), with a maximum frequency offset of Δω = 3 ppm for each z-spectrum. With Δω defined as the specified frequency shift difference, the gagCEST effect was further evaluated using the maximum magnetization transfer asymmetry (MTRasym): MTRasym(Δω) = Z(−Δω) − Z(Δω)) [21]. More specifically, using the average value of MTRasym in the GAG-specific frequency range (Δω = 0.9–1.9 ppm) in which hydroxyl protons of GAG can function as CEST agents [16], MTRasym maps were calculated with MTRasym values provided in %.

To determine the CEST effect, disc segmentation was performed according to the Naïve Bayes classification for image segmentation, which differentiates disc tissue from osseous and ligamentous structures of the lumbar spine [26]. To this end, region-of-interest (ROI) definition was automatically conducted by computed segmentation of lumbar IVDs using a customized in-house script implemented in Matlab with the ROIs subsequently positioned in the lumbar IVDs. A radiologist (CS) visually verified the correct ROI position for every IVD. If necessary, mispositioned ROIs were repositioned, yet no ROI size had to be modified manually. Hereafter, MTRasym values are referred to as gagCEST values for easier readability.

### 2.4. Statistical Analysis

Statistical analyses were performed by KLR and LMW using SPSS (v27, SPSS Inc., Chicago, IL, USA). Summary statistics of gagCEST values were determined for healthy controls and AIS patients. Based on a linear mixed model (LMM), both cohorts (i.e., healthy controls and AIS patients), regions (i.e., NP and AF), and IVD group (i.e., uaIVDs and aIVDs) were comparatively evaluated as multivariable statistics. The LMMs included a subject-specific random intercept, independent variables (i.e., gender, age, cohort, Pfirrmann score, IVD segment level, scoliotic affection), and their interactions. Employing a restricted maximum likelihood approach, the LMMs were fitted [27]. Mean differences of gagCEST values were determined based on the established LMMs and analyzed for significance. Due to this study’s exploratory character, *p*-values ≤ 0.05 indicated statistical significance.

## 3. Results

### 3.1. Study Population

The characteristics of the study population are displayed in Table 1. A total of 80 lumbar IVDs of 16 healthy controls and 50 lumbar IVDs of 10 AIS patients (both L1/L2–L5/S1) were analyzed. For both groups, significant differences in age distribution were found with AIS patients being significantly younger than healthy controls (*p* < 0.001). The covariates were corrected for an age of 23.1 years.

### 3.2. Morphologic Grading of IVD Degeneration

Based on the Pfirrmann classification, degeneration in IVDs was quantified as follows: total study population: grade 2: (*n* = 127), grade 3: (*n* = 3); healthy controls: grade 2: (*n* = 79), grade 3: (*n* = 1); AIS patients: grade 2: (*n* = 48), grade 3: (*n* = 2). No IVD grades 1, 4, or 5 were present. 

### 3.3. GagCEST Values: Healthy Controls vs. AIS Patients

In all lumbar IVDs of both groups, significantly higher gagCEST values were observed in the NP than the AF (NP: 3.90 [confidence interval (CI) 3.49, 4.31]%, AF: 2.37 [CI 2.03, 2.70]%; *p* = 0.001). 

Over all segments, IVDs in AIS patients exhibited significantly lower mean gagCEST values compared to those of healthy controls (AIS: 2.76 [CI 2.32, 3.20]%, C: 3.51 [CI: 3.16, 3.86]%; *p* = 0.005). Considering each lumbar segment by itself, gagCEST values only significantly differed at segment L5/S1 (AIS 1.38 [0.66, 2.1]%, C; 3.39 [2.87, 3.90]%; *p* < 0.001

**Figure 1:**
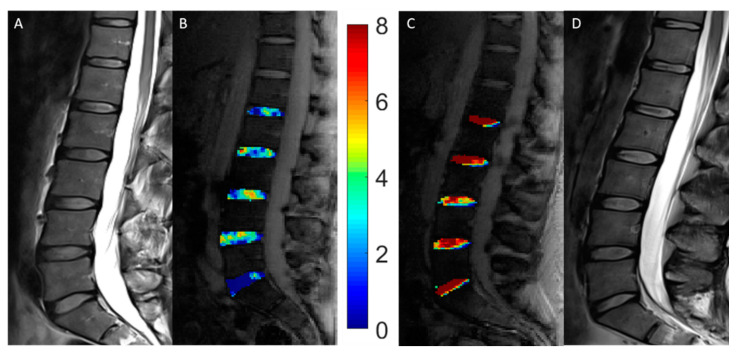
Representative morphologic and compositional imaging findings of the lumbar intervertebral discs (IVDs) of a patient with adolescent idiopathic scoliosis (AIS, **A**,**B**) and a healthy control (**C**,**D**). (**A**,**D**): Sagittal T2-weighted images show the absence of morphologic signs of relevant intervertebral disc degeneration. (**B**,**C**): Sagittal gagCEST images with overlaid color-coded gagCEST maps to indicate the GAG content reveal distinct differences in GAG contents. Lower GAG content is indicated in blue, while high GAG content is illustrated in red. This patient with AIS demonstrated a lower GAG content than this healthy control despite non-degenerated IVDs in both individuals.

Mean gagCEST values in healthy controls and AIS patients as well as the corresponding comparative analysis are further outlined in Table 3. GagCEST maps of both study groups illustrate these findings (Figure 1). 

### 3.4. GagCEST Values: Affected vs. Unaffected IVDs

**Figure 2:**
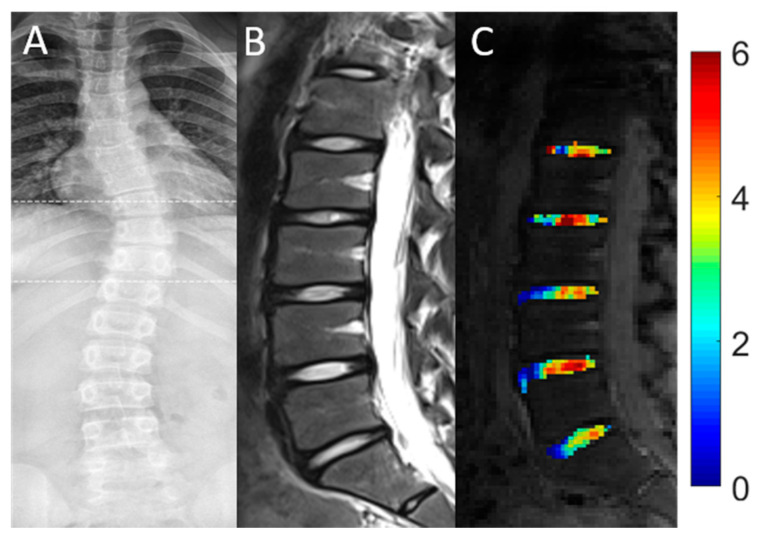
Representative conventional radiograph (**A**) and morphologic (**B**) and compositional (**C**) imaging findings of the spine of a 15-year-old female patient. (**A**): Conventional whole-spine radiograph in coronal orientation reveals the extent of the scoliotic curve. (**B**): Sagittal T2-weighted image indicates the absence of significant degenerative changes of the lumbar IVDs. (**C**): Sagittal gagCEST images with overlaid color-coded gagCEST maps to illustrate the GAG content indicates that no significant differences of the GAG content are seen in scoliotic compared to non-scoliotic IVDs.

In AIS patients, 44% of IVDs (22/50) were found to be affected by scoliotic deformity (aIVDs). Correspondingly, 56% of IVDs (28/50) were identified as unaffected (uaIVDs). The 22 aIVDs in the AIS patients were distributed across the different segment levels as follows: 9 aIVDs were located in segment L1/2, 6 aIVDs in segment L2/3, 4 aIVDs in segment L3/4 and 3 aIVDs in segment L4/5. In segment L5/S1 no IVD was affected by scoliotic deformity. Over all segments, no significant differences were found for mean gagCEST values between uaIVDs and aIVDs (uaIVDs: 2.32 [CI 0.93, 3. 70]%, aIVD: 2.93 [CI 1.48, 4.38]%; *p* = 0.258) as detailed in Table 4. Representative morphological and compositional imaging findings are shown in Figure 2.

## 4. Discussion

The most important finding of this study was that gagCEST values in lumbar IVDs of AIS patients were significantly lower than those of healthy controls, thereby indicating premorphologic IVD degeneration secondary to the specific biomechanics associated with scoliotic curvatures.

GAG depletion is considered the initial, yet reversible, step in cartilage degeneration, e.g., in degenerative disc disease of the lumbar spine [4,17]. GagCEST is a novel imaging technique that enables the quantification of GAG content in IVDs without the application of GBCA, thus rendering feasible the evaluation of early cartilage changes at the premorphologic level [20]. Decreasing gagCEST values are associated with increasing IVD degeneration, as classified using Pfirrmann grading [19,20].

In line with the results of previous studies [4,17,19,20,22], we found that gagCEST values of the NPs in all lumbar IVDs were significantly higher than those of the AF. In healthy IVDs, GAG content is physiologically highest in the NP and lowest in the AF [4,19,20]. Our group previously demonstrated significantly lower gagCEST values in the AF than the NP in a healthy cohort [20]. In a histological study, Urban et al. examined disc composition in patients with neuromuscular scoliosis and found the GAG and water content to be highest in the disc center with a steady decrease towards the periphery, i.e., the NP–AF transition [28]. Accordingly, our study revealed the same basic IVD structure, i.e., an expected GAG gradient from central (NP) to distal (AF), in both healthy controls and AIS patients.

Overall, gagCEST values in lumbar IVDs of AIS patients were significantly lower as compared to those of healthy controls. Lower GAG concentrations bring about a loss in hydration, thus reducing biomechanical resistance [13,24,29]. Previous studies have indicated that such compositional changes in lumbar IVDs occur before morphologic degeneration [14], thereby underscoring the diagnostic potential of gagCEST imaging in the detection of early, premorphologic degeneration. Accordingly, our results suggest that IVDs of AIS patients already suffer from more severe GAG depletion and hence early degenerative changes at a significantly younger age. Once these patients have attained skeletal maturity, their lumbar spines are characterized by ongoing compositional disintegration secondary to their aberrant biomechanics. As these changes may progress further with increasing age [21], thereby underscoring the clinical need to diagnose early degeneration, timely diagnosis is a prerequisite for timely treatment. In severe cases of scoliotic deformity, with a Cobb angle of over 50°, spinal fusion surgery remains the treatment of choice in AIS patients [8,9] after skeletal maturity. As of today, it is still controversially discussed whether spinal fusion in the lower lumbar segments leads to the development of premature disc degeneration and subsequent back pain in AIS patients by reducing the number of mobile segments or if the disease itself might be the cause due to mechanical stress of the spinal curvature [30,31]. However, progression of IVD degeneration has not yet been correlated to Cobb angle. Nonetheless, reducing the number of fixed segments influences the development of disc degeneration and may lead to less lumbar back pain [32]. So far, only a few studies have evaluated the rates of preoperative disc degeneration in the lumbar spines of AIS patients [10]. In a retrospective study, Jones et al. found that only 3.9% of AIS patients undergoing surgical interventions exhibited degenerative disc changes preoperatively, which is comparable to the general pediatric population [10]. With most of the preoperative morphologic MRI scans appearing normal in AIS patients, they concluded that surgical interventions should be aimed at preserving motion in the lumbar spine segments [10]. However, in our study, lower gagCEST values in conservatively treated AIS patients indicate early compositional changes of the IVDs, suggesting that early degenerative disc changes might already be prevalent preoperatively even if not yet visible morphologically. Even though it remains speculative whether the established diagnosis of premorphologic IVD degeneration is truly reversible or alters treatment type and course, the fact that IVD composition may be assessed non-invasively extends and refines the treating physician’s diagnostic armamentarium. Nonetheless, further clinical research is necessary to demonstrate the clinical value of diagnosing early compositional IVD changes by means of gagCEST imaging, for example in the preoperative work-up of AIS patients.

Another important finding of our study was that gagCEST values were not significantly different in those IVDs that were a part of the major scoliotic curve as compared to those that were not. In neuromuscular scoliosis, histologic evaluation revealed the highest GAG contents at the apical IVD, i.e., at the apex of the scoliotic curve, compared to adjacent discs one or two levels below, suggesting increased mechanical load in the convex region of the curve [28]. Increasing mechanical stress applied to the disc induces a decrease in disc hydration, thereby increasing relative GAG content (as a percentage of wet weight) [28]. Regardless of the exact relationship of solid and fluid IVD components, histologic data suggest that decreases or depletion of GAGs are not present in severely scoliotic segments. These results also indicate that aberrant loading, as it occurs in patients with neuromuscular scoliosis, does not directly affect GAG content [28], which is in accordance with our study. Although the exact reason for the preserved GAG contents in lumbar IVDs remains speculative, the AIS patients’ ages need to be considered. At a mean age of 18.3 ± 8.2 years, the AIS patients may be too young to manifest such compositional changes that are commonly considered the result of long-standing biomechanical aberration. However, due to differences in the rate of curve progression, severity of final curvature, and rigidity of the scoliotic deformity [33,34,35], patients with AIS and neuromuscular scoliosis may not be directly compared. Yet, due to the small number of patients with AIS enrolled in our study, our data might only be considered preliminary and should thus be validated in a larger and more homogenous patient cohort.

Our study has limitations. First, the small patient numbers are the main limitation of our study. Even though the analysis included 130 separate IVDs, these were necessarily interrelated. Consequently, with *n* = 10 AIS patients included, our results can only be considered preliminary. However, this is the first study using gagCEST imaging for the compositional evaluation of lumbar IVDs in patients with AIS that could be a starting point for future larger studies. Second, our study population was not age matched. The significantly lower mean age in AIS patients may potentially have affected our results since GAG content is known to decrease with increasing age [21]. Yet, in favor of our central hypothesis, we still found significantly lower gagCEST values in AIS patients, who, because of their younger age, ought to have higher GAG values than in healthy controls. Since the onset of AIS most commonly manifests in early puberty, a very young patient cohort was expected [1,2]. Due to ethical reasons, however, MRI exams of minors as healthy age-matched controls could not be performed. However, the significant difference in age distribution of both cohorts may be considered of minor relevance since (i) the factor “age” was accounted for in the LMM and (ii) age itself is a risk factor for GAG depletion. Third, the imaging data could not be validated through reference analyses, e.g., by histology, for ethical reasons. Fourth, evaluation of GAG content was only performed in lumbar IVDs. Though thoracic IVDs are often more severely affected by scoliotic deformity and thoracic scoliotic deformities tend to progress more rapidly [36], we only evaluated GAG content in lumbar IVDs, as thoracic IVDs are prone to considerable motion artifacts caused by respiratory motion. Fifth, the severity of scoliotic deformity, as indicated by the Cobb angle, was quite heterogeneous among our AIS patients, ranging from 11° to 73°, which may explain increased statistical variability, yet only demonstrates clinical reality. Still, future studies should include a larger study population that accounts for the various clinical and diagnostic factors such as disease duration, degree of curve deformity, and shape and degree of the Cobb angle by large enough patient numbers and sufficient statistical power. 

## 5. Conclusions

In conclusion, the lumbar IVDs of adolescent and adult AIS patients revealed significantly lower gagCEST values indicative of compositional proteoglycan depletion that precedes structural changes and were not (yet) visible morphologically. Thus, as a powerful non-invasive and contrast-agent-free diagnostic adjunct, gagCEST imaging may be prospectively applied to quantify IVD composition and to detect early compositional changes of the degenerative cascade in patients affected by AIS. 

## Figures and Tables

**Table 1:** Demographic, clinical, and therapeutic information of the study population.

	AIS Patients	Controls
**Age [years]**	18.3 ± 8.2	25.5 ± 1.7
**Gender [female/male]**	7/4	8/8
**Number of involved vertebrae [*n*]**		
● Major curve	7.5 ± 3.2 (4–15)	n/a
● Minor curve	6 ± 0.5 (5–6)	n/a
**Orientation of major curve [dextro-, levoscoliosis]**	Dextroscoliosis *n* = 6 Levoscoliosis *n* = 5	n/a
**Cobb angle [°] major curve**	34.8 ± 21.5 (15–73)	n/a
**Cobb angle [°] minor curve**	21.8 ± 10.2 (11–37)	n/a
**Disease duration [months]**	59.2 ± 102.0	n/a
**Type of treatment**		n/a
● Physical therapy	4	n/a
● Bracing (conservative)	5	n/a
● Bracing (+subsequent surgery)	2	n/a

Data for the radiographic analysis (Cobb angle of the major and minor curve, the number of involved vertebrae, the orientation of the major curve, and the disease duration) and type of treatment are given for AIS patients. Means ± standard deviations and the range are presented. Abbreviations: AIS—adolescent idiopathic scoliosis; n/a—not applicable.

**Table 2:** Morphological and compositional magnetic resonance imaging (MRI) sequence parameters.

	Sequence				
	STIR	T2w TSE	T1w TSE	gagCEST	WASSR
**Orientation**	Sagittal	Sagittal	Sagittal	Sagittal	Sagittal
**TE/TR [ms]**	57/3800	95/3500	9.5/650	5.1/10	5.1/10
**Flip Angle [°]**	150	160	150	10	10
**Slice Thickness [mm]**	4	4	4	5	5
**FoV [mm × mm]**	300 × 300	300 × 300	300 × 300	300 × 300	300 × 300
**Pixel Size [mm × mm]**	0.8 × 0.8	0.7 × 0.7	0.7 × 0.7	1.6 × 1.6	1.6 × 1.6

Orientation, echo and repetition time, flip angle, slice thickness, field of view, pixel size, and number of slices are given for all sequences. Abbreviations: TE—echo time; TR—repetition time; FOV—field of view; STIR—short tau inversions recovery; T2w—T2-weighted; T1w T2-weighted; TSE—turbo spin-echo; gagCEST—glycosaminoglycan chemical exchange saturation transfer; WASSR—water saturation shift referencing.

**Table 3:** Analysis of compositional imaging features of lumbar intervertebral discs (IVDs).

Score		L1/2	L2/3	L3/4	L4/5	L5/S1	All Segments
**gagCEST values [%]**	**Controls**	3.340 [2.721, 3.959]	3.466 [2.844, 4.089]	3.218 [2.586, 3.850]	3.679 [2.082, 5.276]	3.389 [2.877, 3.902]	3.510 [3.159, 3.861]
	**AIS**	2.867 [2.005, 3.729]	2.646 [1.831, 3.461]	3.908 [3.079, 4.736]	3.700 [1.581, 5.818]	1.381 [0.661, 2.102]	2.759 [2.320, 3.199]
	***p*-values**	0.399	0.139	0.220	0.988	<0.001	0.005

Mean glycosaminoglycan chemical exchange saturation transfer (gagCEST) values of the two study cohorts (i.e., 16 healthy controls and 10 AIS patients) in all patients as a function of segment levels. Using linear mixed models including a subject-specific intercept, mean gagCEST values were compared. Data are corrected mean + 95% confidence interval [square brackets] (%) of gagCEST values of the nucleus pulposus (NP). The covariates were corrected for an age of 23.1 years. *p*-values < 0.05 are given in bold type. Abbreviations: L—lumbar; n/a—not applicable.

**Table 4:** Analysis of scoliotic affection on compositional imaging features of lumbar intervertebral discs (IVDs) in AIS patients. Mean glycosaminoglycan chemical exchange saturation transfer (gagCEST) values as a function of scoliotic affection (affected and unaffected IVDs of AIS patients) and IVD segment levels. Using a linear mixed model including a subject-specific intercept, mean values of gagCEST were compared. Data are corrected mean + 95% confidence interval [square brackets] (%) of gagCEST values of the nucleus pulposus (NP). The covariates were corrected for an age of 23.1 years. *p*-values < 0.05 were considered significant. Abbreviations: L—lumbar; n/a—not applicable.

Score	Segment							*p*-Value
		L1/2	L2/3	L3/4	L4/5	L5/S1	All Segments	
**IVDs** **affected** **by scoliosis**	**Yes**	3.171 [0.129, 4.213]	2.886 [2.155, 3.616]	5.103 [2.686, 7.520]	1.520 [0.377, 2.663]	n/a	2.929 [1.479, 4.379]	0.258
	**No**	−0.004 [−3.666, 3.659]	2.666 [1.179, 4.153]	3.100 [2.093, 4.107]	5.762 [1.116, 10.408]	2.186 [0.942, 3.429]	2.316 [0.936, 3.697]	

## Data Availability

The data presented and/or analyzed in this study are available on reasonable request from the corresponding author. The data are not publicly available due to ethical reasons.
